# Comorbidity network analysis using graphical models for electronic health records

**DOI:** 10.3389/fdata.2023.846202

**Published:** 2023-08-17

**Authors:** Bo Zhao, Sarah Huepenbecker, Gen Zhu, Suja S. Rajan, Kayo Fujimoto, Xi Luo

**Affiliations:** ^1^Department of Biostatistics and Data Science, School of Public Health, The University of Texas Health Science Center, Houston, TX, United States; ^2^Department of Gynecologic Oncology and Reproductive Medicine, The University of Texas MD Anderson Cancer Center, Houston, TX, United States; ^3^Department of Management, Policy and Community Health, School of Public Health, The University of Texas Health Science Center, Houston, TX, United States; ^4^Department of Health Promotion and Behavioral Sciences, School of Public Health, The University of Texas Health Science Center, Houston, TX, United States

**Keywords:** comorbidity network analysis, graphic modeling method, machine learning, electronic health records, critical care unit

## Abstract

**Importance:**

The comorbidity network represents multiple diseases and their relationships in a graph. Understanding comorbidity networks among critical care unit (CCU) patients can help doctors diagnose patients faster, minimize missed diagnoses, and potentially decrease morbidity and mortality.

**Objective:**

The main objective of this study was to identify the comorbidity network among CCU patients using a novel application of a machine learning method (graphical modeling method). The second objective was to compare the machine learning method with a traditional pairwise method in simulation.

**Method:**

This cross-sectional study used CCU patients' data from Medical Information Mart for the Intensive Care-3 (MIMIC-3) dataset, an electronic health record (EHR) of patients with CCU hospitalizations within Beth Israel Deaconess Hospital from 2001 to 2012. A machine learning method (graphical modeling method) was applied to identify the comorbidity network of 654 diagnosis categories among 46,511 patients.

**Results:**

Out of the 654 diagnosis categories, the graphical modeling method identified a comorbidity network of 2,806 associations in 510 diagnosis categories. Two medical professionals reviewed the comorbidity network and confirmed that the associations were consistent with current medical understanding. Moreover, the strongest association in our network was between “poisoning by psychotropic agents” and “accidental poisoning by tranquilizers” (logOR 8.16), and the most connected diagnosis was “disorders of fluid, electrolyte, and acid–base balance” (63 associated diagnosis categories). Our method outperformed traditional pairwise comorbidity network methods in simulation studies. Some strongest associations between diagnosis categories were also identified, for example, “diagnoses of mitral and aortic valve” and “other rheumatic heart disease” (logOR: 5.15). Furthermore, our method identified diagnosis categories that were connected with most other diagnosis categories, for example, “disorders of fluid, electrolyte, and acid–base balance” was associated with 63 other diagnosis categories. Additionally, using a data-driven approach, our method partitioned the diagnosis categories into 14 modularity classes.

**Conclusion and relevance:**

Our graphical modeling method inferred a logical comorbidity network whose associations were consistent with current medical understanding and outperformed traditional network methods in simulation. Our comorbidity network method can potentially assist CCU doctors in diagnosing patients faster and minimizing missed diagnoses.

## Introduction

Diseases can share similar genetic and environmental causes and may correlate with each other. Comorbidity is the concurrent presence of two or more diseases or medical conditions in the same patient. A graphical approach called comorbidity network analysis (CNA) is usually employed to study many comorbidities together and discover the associations between diseases in a more realistic sense (Cramer et al., 2010).

CNA is important since it helps us to understand disease correlations from a broader perspective, and sometimes, it can even reveal novel correlations for further investigation (Hidalgo et al., 2009). Compared with the latent variable approach, CNA generates a representation of candidate pathways between comorbidities (Cramer et al., 2010; Eaton, 2015). It can inform doctors of a patient's possible comorbidities given his primary disease, helping doctors diagnose their patient faster and minimize missed diagnoses. In fact, CNA has been used to identify the correlation between diseases and has mainly been applied to chronic diseases (Khan et al., 2018; Aguado et al., 2020; Cruz-Ávila et al., 2020; Sriram et al., 2021). For example, the comorbidity network of type 2 diabetes was analyzed by Aguado et al. (2020). In 2021, Sriram et al. also analyzed the comorbidity network among women with obstetric disorders from UK Biobank (Sriram et al., 2021).

Fotouhi et al. (2018) summarized statistical methods that were used to construct the comorbidity network. Though with different metrics (OR, RR, or phi), these methods all used a pairwise relationship to build the comorbidity network. Recently, pairwise mutual information with permutation test inference was employed to study type 2 diabetes risk factors and comorbidity network modules (Preo and Capobianco, 2019). The drawback of a pairwise method was that it did not control for other potentially confounding diseases and might recover superficial or indirect associations between diseases. For example, from a clinical perspective, obesity causes diabetes and coronary artery disease. If left uncontrolled, diabetes can cause diabetic foot ulcers. If coronary artery disease and diabetic foot ulcer are studied pairwise, a superficial relationship exists since they share a common cause (obesity). However, if obesity or diabetes or both are controlled, then this indirect association between coronary artery disease and diabetic foot ulcers will disappear.

On the other hand, the introduction of electronic health records (EHR) brings more challenges to the current method because we have access to many more covariates and observations. However, many of the covariates might be irrelevant to the outcome, if we include all covariates, unnecessary complexity will be added to our model, which may create an over-fitting problem, and the model could have low external generalizability in real-world clinical practice.

To handle these problems, traditional statistics use iterative variable selection methods, either forward, backward, or stepwise selection. However, these iterative selection algorithms might drop significant but correlated covariates because multicollinearity will inflate the variance dramatically. To avoid this drawback, an exhaustive search can be utilized by comparing all possible models. However, it is computationally prohibitive as one needs to search among all possible models: when p becomes large, for example, 500, then 3.27^*^10^150^ models are needed to be fit, which is computationally impossible. Based on these disadvantages, a new statistical method is needed to control for confounders and be efficient in model selection.

In this study, we aim to apply a graphical modeling method (Dalege et al., 2017; Epskamp et al., 2018) to construct a full-scale comorbidity network from HER data. This differs from state-of-the-art pairwise methods, where our proposal aims to recover conditional dependences between diseases that are more likely to be direct connections. Motivated by the sparse characteristics of EHR data, we also adapt and compare different choices of penalty for network estimation using extensive simulation studies. This leads to an improved method suitable for EHR analysis. Finally, we assess the validity of our proposal using a real data analysis validated by two medical professionals. As far as we are aware, this is among the first applications of graphical modeling to EHR data. In the Method section, we first describe the MIMIC-3 EHR dataset and then introduce our proposal on how to adapt the graphical modeling method for the EHR setting. We also describe the simulation study to evaluate the validity of our proposal and compare its performance with the existing pairwise approach. In the Result section, we present the comorbidity network recovered from analyzing a critical care unit (CCU) database. These network connections between diseases were reviewed and validated by two medical professionals. We also present the simulation results showing the validity of our proposal and its improvement over the existing approach. Finally, the Discussion section presents the implications and limitations of our results.

## Method

### Study dataset

**Figure 1:**
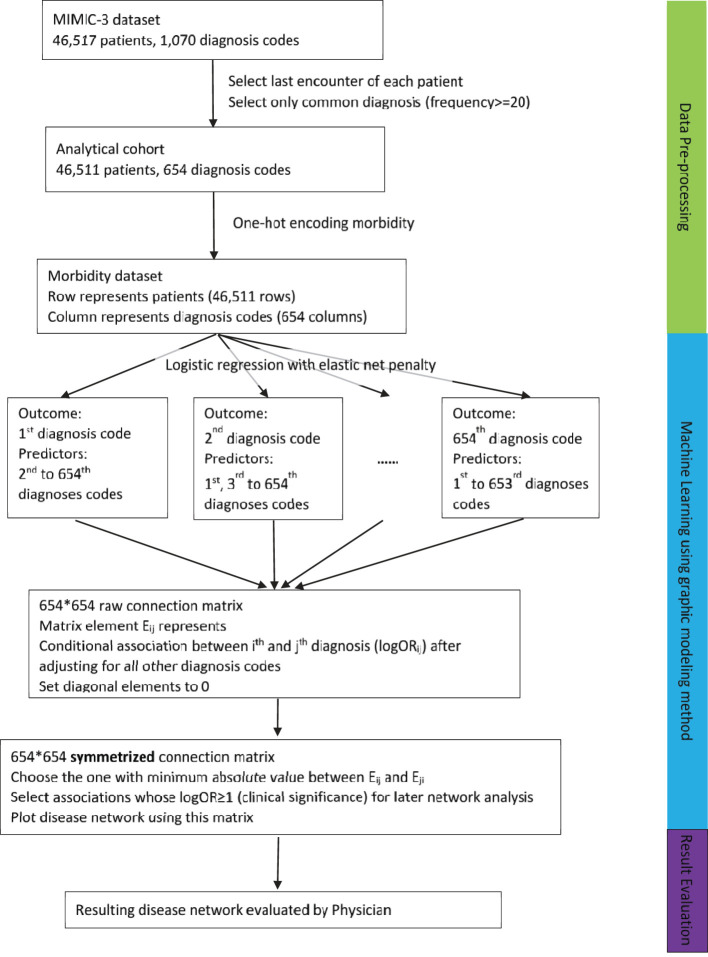
Workflow of our data processing, analysis, and evaluation steps.

The Medical Information Mart for Intensive Care-3 (MIMIC-3) datasets (Johnson et al., 2016) were used in this study, which are encounter datasets of patients who stayed in the CCUs of the Beth Israel Deaconess Hospital from 2001 to 2012. Most patients (83%) had only one encounter. However, if a patient had multiple encounters, only the last encounter of the patient was kept to generate an independent sample. The workflow of the study process can be found in Figure 1, including the data pre-processing and the analysis process.

### Morbidity identification

Patients' general diagnosis categories were identified by ICD-9 codes. Following ICD-9 coding syntax (List of ICD-9 codes, 2022) for ICD-9 codes starting with numbers, three-digit codes were used to determine the patients' general diagnosis category. For ICD-9 codes starting with “V” or “E,” two digits after “V” and three digits after “E” was used. After consolidating ICD-9 codes into general categories, duplicates of ICD-9 codes within the same encounter were removed. To only emphasize common diagnosis categories, we excluded diagnosis categories whose frequencies were < 20 in the sample. This helped improve the fit of the statistical model by excluding the outcomes that were too less (sparse). The number 20 was selected as a rule of thumb. Each encounter was then double-checked to ensure it had at least one diagnosis after this step.

### Morbidity dataset

Within each encounter, a series of binary dummy variables were created for all general diagnosis categories, indicating whether or not the encounter had the diagnosis or not. In this way, a morbidity dataset was created, with columns representing diagnosis categories (*P*) and rows representing patients (*N*).

### Statistical analysis

The graphical modeling method (Strobl et al., 2012) was used to model the data. It was modified to fit EHR settings by replacing the LASSO penalty with the elastic net penalty (Zou and Hastie, 2005). The algorithm iterated over each of the diagnosis categories as follows: at iteration *i*, the *i*th diagnosis category was selected as the outcome, and a logistic regression with the elastic net penalty was fitted on all other diagnosis categories as covariates. The resulting coefficients (logOR) were put into the *i*th row of the *P*×*P* weight matrix without the diagonal entry. The above process was repeated until all disease categories had been chosen as outcomes.

**Figure 2:**
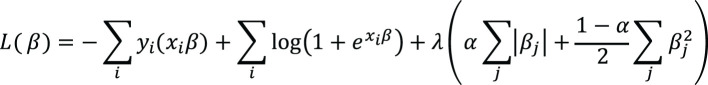
The penalized logistic regression loss with the elastic net penalty. The first two terms are the regular negative log-likelihood function of logistic regression. The third term is the elastic net penalty function. The third term: λ: penalty coefficient, chosen by eBIC for best model fit. α: elastic net tuning parameter, deciding the percentage of LASSO vs. Ridge in the penalty term. $\overset{p}{\underset{j=1}{\sum }}|\beta_{j}|$: LASSO penalty. $\overset{p}{\underset{j=1}{\sum }}\beta_{j}^{2}$: ridge penalty.

For the *P*×*P* weight matrix, each off-diagonal element (logOR_ij_) represented the associations between diagnosis category *i* and diagnosis category *j* in the form of a log odds ratio. Due to the elastic net penalty, logOR_ij_ for some (*i, j*) pairs was set to 0, indicating zero or undetectable associations. The likelihood function of the elastic net is shown below (Zou and Hastie, 2005) (Figure 2).

The existing graphical modeling method for binary data (Strobl et al., 2012) used the LASSO penalty, which corresponds to a special case of α = 1 in the likelihood. Elastic net usually outperforms LASSO on data with high collinearity, and this motivated us to adopt the elastic net penalty for EHR data. In this weight matrix, logOR_ij_ and logOR_ji_ at symmetric positions had different meanings: logOR_ij_ is the log of the odds ratio of disease category i and j, setting i as the outcome while j as a covariate in the logistic model, after adjusting for all other diseases categories. In contrast, logOR_ji_ was the log of the odds ratio of disease category j and i, setting j as the outcome while i as a covariate in the logistic model after adjusting for all other disease categories.

To be more conservative, for logOR_ij_ and logOR_ji_ at symmetric positions in the matrix, we selected the one with a minimum absolute value and replaced the other value with it. In this way, a symmetric matrix was constructed to represent an undirected, weighted disease network. This disease network was then plotted using Gephi (Bastian et al., 2009). The graph layout was “Force Atlas,” with repulsion strength = 10,000. The size of each node represented its degree, and the color of each node represented its modularity class. The resulting comorbidity network was then reviewed by two medical professionals (Dr. Sarah Huepenbecker and Dr. Bo Zhao).

For comparison, the pairwise OR method (Aguado et al., 2020) was also applied in the dataset as follows. For each pair of diagnosis categories, a 2 by 2 contingency table was constructed first. The odds ratio (OR) and *P*-value from Fisher's exact test were calculated (Rosner, 2010) to assess the associations between the diseases. This process was repeated for all pairs of diagnosis categories. Then, the *P*-value is adjusted using the Bonferroni correction. The ORs whose adjusted *P*-values were < 0.05 were kept. Unlike our proposal, the pairwise method did not adjust for all other diseases to assess the pairwise associations between diseases.

### Outcomes

The primary outcome of our analysis method was a *P*×*P* network weight matrix, with off-diagonal elements representing the associations between different diagnosis categories in terms of the log odds ratio, after correcting for all other diseases. Graph theoretical statistics, including edge density, global transitivity, diameter, and average distance, were used to summarize the overall characteristics of the recovered network. The weight matrix was then studied to find the strongest associations, which were denoted as the top edges, and diagnosis categories that were associated with most other diagnosis categories were also examined, which were denoted as the top nodes. The weight matrix was also converted to a 0/1 adjacency matrix for further unweighted analysis. For the top nodes, both unweighted and weighted results were presented. The difference between the two was whether the connections were treated as binary values (0 or not 0) or not when calculating for the top nodes.

For further exploration of the recovered network, the diagnosis categories were partitioned into different subgroups based on their associations with other diagnosis categories. If some diagnosis categories were closely associated, they were partitioned into one subgroup. The subgroups were called modularity classes.

### Simulation validation

Our proposed method was compared with traditional methods in six different scenarios. Two node-sample combinations were generated: 100 nodes 5k sample size and 100 nodes 10k sample size. Within each node-sample combination, we simulated three sparse rates (probability of the existence of an edge): 0.05, 0.1, and 0.2. This led to a total of six different scenarios, and they were listed as follows:

1) 100 nodes, 5k sample size, 0.05 sparse rate,
2) 100 nodes, 5k sample size, 0.1 sparse rate,
3) 100 nodes, 5k sample size, 0.2 sparse rate,
4) 100 nodes, 10k sample size, 0.05 sparse rate,
5) 100 nodes, 10k sample size, 0.1 sparse rate, and
6) 100 nodes, 10k sample size, 0.2 sparse rate.

The simulation method for each scenario is described below:

1) Generate a network weight matrix of a random network with a given number of nodes and sparse rate. It is named as the “original weight matrix.”
2) Given the “original weight matrix” and sample size, simulate correlated binary data with the Metropolis–Hastings algorithm (Hastings, 1970) using the R package IsingSampler. The columns represent the nodes, while the rows represent samples.
3) Given the simulated data, calculate the weight matrices using either the traditional method (pairwise OR calculated from a 2 by 2 contingency table) or the graphic modeling methods with different percentages of LASSO penalty (100%, 90%, and 70%). They are named as “estimated weight matrices.”
4) Compare “estimated weight matrices” with the “original weight matrix” and evaluate which method can output a weight matrix closer to the original one. Evaluation metrics include Euclidean distance, Spectral distances, and NetSimile (Tantardini et al., 2019).

Pairwise mutual information networks with permutation-based inference (Preo and Capobianco, 2019) require high computational costs for large networks. We thus compared with mutual information networks using small network simulations with 20 nodes and 5k sample size, while all the other simulation procedures were kept the same.

## Result

### Study population

The MIMIC dataset included 46,517 patients, 58,929 encounters, and 1,070 three-digits ICD-9 diagnosis categories in the MIMIC dataset. The majority of the patients (38,609; 83%) had only one encounter. After selecting the last encounter for each patient, and only common diagnosis categories (frequency ≥ 20), 46,511 patients/encounters and 654 diagnosis categories were included in our analysis.

The patient's demographics are presented in Table 1. Among the patients, 20,395 (43.8%) were women, and 26,116 (56.2%) were men. The median age was 61 years, with an interquartile range of 39–76. In total, 32,434 (69.7%) patients were white, while 3,870 (8.3%) were Black. Medicare (20,926; 45.0%) and private insurance (19,157; 41.2%) were the two major insurance forms.

**Table 1:** Demographic summary statistics of participants included in the analysis.

**Patients demographics-count (%)**
**Gender**	
Female	20,395 (43.8%)
Male	26,116 (56.2%)
Age-median (IQR)	61 (39–76)
**Race/ethnicity**	
White	32,434 (69.7%)
Black	3,870 (8.3%)
Hispanic	1,641 (3.5%)
Asian	1,690 (3.6%)
Other/unknown	6,876 (14.8%)
**Marital status**	
Married	18,489 (39.8%)
Single	9,766 (21.0%)
Widowed	5,461 (11.7%)
Divorced or separated	2,713 (5.8%)
Other/unknown	10,082 (21.7%)
**Insurance**	
Medicare	20,926 (45.0%)
Private	19,157 (41.2%)
Medicaid	4,369 (9.4%)
Other	2,059 (3.2%)

The median of diagnosis categories per encounter was 8, and the interquartile range was 6 to 13. The top five most prevalent diagnosis categories were essential hypertension (16,791; 36%), cardiac dysrhythmias (13,008; 28%), disorders of lipoid metabolism (11,302; 24%), other forms of chronic ischemic heart disease (11,118; 24%), and disorders of fluid, electrolyte, and acid–base balance (10,869; 23%). A distribution table of all 654 diagnosis categories can be found in Supplementary material 1.

### Comorbidity network presentation

**Figure 3:**
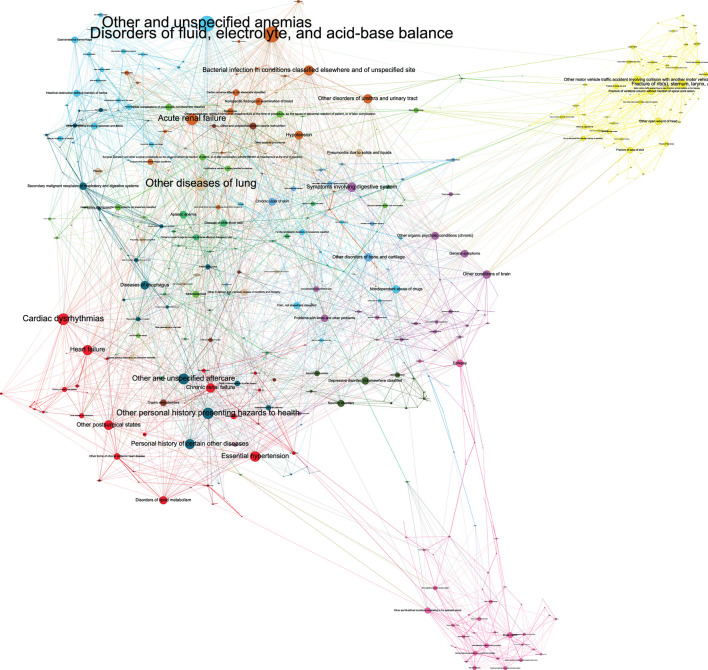
Graph representation of the comorbidity network by our graphical modeling method. The network summary statistics and the interpretation of the top network features (edges, nodes, and modularity classes) were discussed in the main text. The disease network was shown in this figure composed by Gephi12. The graph layout was “Force Atlas,” with repulsion strength = 10,000. The size of node represented its degree and color of node represented its modularity class. The graph was partitioned into 14 modularity classes. The biggest class are the yellow nodes located in the far upper right of the graph. They are diseases related with injury and accident. Some representative diseases with high degree are “Fracture of rib(s), sternum, larynx, and trachea,” “Other motor vehicle traffic accident involving collision with another motor vehicle,” and “Fall on same level from slipping, tripping, or stumbling.” The second biggest class are the blue nodes located in the upper left of the graph. They are diseases related with digestive system. Some representative diseases with high degree are “Intestinal obstruction without mention of hernia,” “Gastrointestinal hemorrhage,” “Peritonitis” and “Chronic liver disease and cirrhosis.” One disease with highest degree is “Other and unspecified anemias,” and it is strongly associated with gastrointestinal hemorrhage, and the model include it in this class. The third biggest class are the pink nodes located in the far lower right of the graph. They are related with neonatal diseases. Some representative diseases with high degree are “Epilepsy,” “Disorders relating to short gestation and unspecified low birthweight,” and “Other perinatal jaundice.” Other classes are related with heart diseases (red nodes in the lower left), renal diseases (brown nodes in the upper middle), pulmonary diseases (light brown nodes in the upper middle), mental diseases (dark green nodes in the lower right), and so on.

Of the 654 diagnosis categories, the graphical modeling method identified 2,806 associations (edges) among 510 diagnosis categories (nodes). The comorbidity network was illustrated in Figure 3. The edge density of the network was 0.02, the global transitivity was 0.24, the diameter was 10, and the average distance was 3.63. A complete list of all 2,806 edges among 510 diagnosis categories, as well as histograms representing the distributions of degree and distance, are provided in Supplementary material 2–4. Interpreting the full details of this network is challenging. Our discussions in the following sections focused on the top network features including edges, nodes, and modularity classes. In brief, in the context of critical care, these top features implicated the significant involvement of diagnoses pertaining to poisoning, diabetes, accidents, as well as the digestive, renal, and respiratory systems.

### Top edges/associations

The top 10 positive and negative edges are presented in Table 2. Of the diagnosis categories included in our analysis, those with the highest positive edges indicate direct “cause-and-effect” relationships after expert review. The two diagnosis categories with the highest positive edges were “poisoning by psychotropic agents” with “accidental poisoning by tranquilizers” (logOR 8.16). Interestingly, three of the top associations involved poisoning. In contrast, two top associations involved foreign bodies in the pharynx and larynx, and two top associations involved disorders related to short gestation. Other highly correlated diagnosis categories included “secondary diabetes mellitus” with “hormones and synthetic substitutes causing adverse events in therapeutic use” (logOR 6.09) and “dermatitis due to substances taken internally” with “antibiotics causing adverse events in therapeutic use” (log OR 5.16). In contrast, the diagnosis categories with the lowest negative edges, indicating mutually exclusive events, most often included single vs. multiple live births and mutually exclusive poisoning, fall, foreign body, and motor vehicle events (Table 2).

**Table 2:** Top edges/connections between diagnoses by weight (logOR) from our graphical modeling method.

**Rank**	**Weight**	**Diagnosis 1**	**Diagnosis 2**
**Top positive edges**			
1	8.16	Poisoning by psychotropic agents	Accidental poisoning by tranquilizers
2	7.59	Foreign body in pharynx and larynx	Inhalation and ingestion of other object causing obstruction of respiratory tract or suffocation
3	7.22	Poisoning by psychotropic agents	Suicide and self-inflicted poisoning by solid or liquid substances
4	7.20	Foreign body in trachea, bronchus, and lung	Inhalation and ingestion of other object causing obstruction of respiratory tract or suffocation
5	6.42	Disorders relating to short gestation and unspecified low birthweight	Other multiple, mates all liveborn
6	6.09	Secondary diabetes mellitus	Hormones and synthetic substitutes causing adverse effects in therapeutic use
7	5.65	Poisoning by primarily systemic agents	Suicide and self-inflicted poisoning by solid or liquid substances
8	5.58	Disorders relating to short gestation and unspecified low birthweight	Twin, mate liveborn
9	5.45	Septicemia	Certain adverse effects, not elsewhere classified
10	5.16	Dermatitis due to substances taken internally	Antibiotics causing adverse effects in therapeutic use
**Top negative edges**			
1	−7.38	Single liveborn	Twin, mate liveborn
2	−5.41	Twin, mate liveborn	Other multiple, mates all liveborn
3	−5.10	Single liveborn	Other multiple, mates all liveborn
4	−4.63	Suicide and self-inflicted poisoning by solid or liquid substances	Poisoning by solid or liquid substances, undetermined whether accidentally or purposely inflicted
5	−4.59	Fall on same level from slipping, tripping, or stumbling	Other and unspecified fall
6	−4.35	Foreign body in pharynx and larynx	Foreign body in trachea, bronchus, and lung
7	−4.23	Fall on or from stairs or steps	Other and unspecified fall
8	−3.94	Fall on or from stairs or steps	Fall on same level from slipping, tripping, or stumbling
9	−3.48	Other motor vehicle traffic accident involving collision with another motor vehicle	Fall on or from stairs or steps
10	−3.25	Other motor vehicle traffic accident involving collision with another motor vehicle	Motor vehicle traffic accident due to loss of control, without collision on the highway

Since negatively correlated diagnosis categories were often mutually exclusive, aiming for clinical significance, we focused on positive associations (logOR) that were not < 1 for the following analysis, which was 2,590 edges among 509 diagnosis categories.

### Top nodes/diagnosis categories

Table 3 provides the top nodes or diagnosis categories that were connected with most other diagnosis codes. Unweighted, the top node was “disorders of fluid, electrolyte, and acid–base balance” (63 associated diagnosis categories), within which the most commonly associated diagnosis categories were “acute renal failure” (logOR 1.09), “disorders of mineral metabolism” (1.08), and “other and unspecified protein-calorie malnutrition” (0.46). “Other and unspecified anemias” was the second highest unweighted node (62 associated diagnosis categories), and “other diseases of lung” (59 associated diagnosis categories) was the third highest unweighted node. Within “other and unspecified anemias,” the most commonly associated diagnosis categories were “gastric ulcer” (0.88), “duodenal ulcer” (0.88), and “gastrointestinal hemorrhage” (0.73); within “other disease of the lung,” the most commonly associated diagnosis categories were “other bacterial pneumonia” (1.53), “pneumonitis due to solids and liquids (1.16),” and “other disease of respiratory system” (1.10).

**Table 3:** Top nodes or diagnoses by unweighted/weighted degree.

**Rank**	**By unweighted degree**	***N* associated diseases**	**By weighted degree**	**N associated diseases**
1	Disorders of fluid, electrolyte, and acid-base balance	63	Other motor vehicle traffic accident involving collision with another motor vehicle	32
2	Other and unspecified anemias	62	Fracture of base of skull	25
3	Other diseases of lung	53	Fracture of rib(s), sternum, larynx, and trachea	35
4	Cardiac dysrhythmias	46	Secondary malignant neoplasm of respiratory and digestive systems	30
5	Acute renal failure	46	Fall on or from stairs or steps	17
6	Other personal history presenting hazards to health	45	Disorders relating to short gestation and unspecified low birthweight	18
7	Other and unspecified aftercare	43	Suicide and self-inflicted poisoning by solid or liquid substances	9
8	Essential hypertension	41	Other and unspecified fall	19
9	Bacterial infection in conditions classified elsewhere and of unspecified site	40	Motor vehicle traffic accident involving collision with pedestrian	17
10	Heart failure	40	Single liveborn	23

When the weighted matrix was used, the top nodes most commonly included those related to injury and accidents. The top node was “other motor vehicle traffic involving a collision with another motor vehicle” (35 associated diagnosis categories), within which “other and unspecified intracranial hemorrhage following injury” (2.04), “injury to spleen” (1.86), and “fracture of vertebral column with spinal cord lesion” (1.75) were the most commonly associated diagnosis categories. Other top weight nodes included “fracture of base of skill” (29 associated diagnosis categories) and “fracture of ribs, sternum, larynx, and trachea” (26 associated diagnosis categories).

### Modularity

The graph was partitioned into 14 modularity classes (Figure 3). The biggest modularity class (yellow nodes in the top right) represented diagnosis categories related to injury and accident, followed by diagnosis categories related to the digestive system and diagnosis categories related to neonatal diseases (pink nodes in the bottom right). The top associated diagnosis categories for the injury and accident, cardiac, and neonatal modularity classes are shown in Table 4. For example, within the cardiac modularity, the most commonly associated diagnosis categories were “diseases of mitral and aortic valve” with “other rheumatic heart disease” (logOR 5.15), with other common associations related to chronic ischemic disease and renal hypertensive disease codes. Within the neonatal modularity, the most commonly associated diagnosis categories included short gestation and intellectual disorders, with “disorders relating to short gestation and unspecified low birth rate” with “other multiple, mates all liveborn” (6.42) as the most commonly associated diagnosis categories. Within the accident and trauma modularity, common associations included unspecified falls, intracranial hemorrhage, and fractures.

**Table 4:** Top connections by modularity class.

**Cardiac**			**Neonatal**			**Injury and accident**		
**Diagnosis 1**	**Diagnosis 2**	**Weight**	**Diagnosis 1**	**Diagnosis 2**	**Weight**	**Diagnosis 1**	**Diagnosis 2**	**Weight**
Diseases of mitral and aortic valves	Other rheumatic heart disease	5.15	Disorders relating to short gestation and unspecified low birthweight	Other multiple, mates all liveborn	6.42	Subarachnoid, subdural, and extradural hemorrhage, following injury	Other and unspecified fall	4.75
Diseases of mitral valve	Other rheumatic heart disease	4.8	Disorders relating to short gestation and unspecified low birthweight	Twin, mate liveborn	5.58	Subarachnoid, subdural, and extradural hemorrhage, following injury	Fall on same level from slipping, tripping, or stumbling	4.44
Need for isolation and other prophylactic measures	Personal history of allergy to medicinal agents	4.39	Unspecified intellectual disabilities	Infantile cerebral palsy	4.38	Subarachnoid, subdural, and extradural hemorrhage, following injury	Fall on or from stairs or steps	4.3
Hypertensive renal disease	Chronic renal failure	4.07	Need for other prophylactic vaccination and inoculation against single diseases	Single liveborn	4.09	Contusion of upper limb	Agents primarily acting on the smooth and skeletal muscles and respiratory system causing adverse effects in therapeutic use	3.9
Other acute and subacute form of ischemic heart disease	Other forms of chronic ischemic heart disease	3.15	Other specified intellectual disabilities	Spinocerebellar disease	3.61	Fracture of neck of femur	Fall on same level from slipping, tripping, or stumbling	3.89
Hypertensive heart and renal disease	Chronic renal failure	2.95	Observation and evaluation of newborns and infants for suspected condition not found	Single liveborn	3.53	Fracture of pelvis	Dislocation of hip	3.67
Family history of certain chronic disabling diseases	Family history of certain other specific conditions	2.92	Other specified intellectual disabilities	Infantile cerebral palsy	3.26	Fracture of tibia and fibula	Motor vehicle traffic accident involving collision with pedestrian	3.53
Diseases of mitral and aortic valves	Diseases of other endocardial structures	2.91	Other specified intellectual disabilities	Chromosomal anomalies	2.88	Other, multiple, and ill-defined dislocations	Spinal cord injury without evidence of spinal bone injury	3.47
Angina pectoris	Other forms of chronic ischemic heart disease	2.73	Dentofacial anomalies, including malocclusion	Certain congenital musculoskeletal deformities	2.84	Contusion of eye and adnexa	Contusion of upper limb	3.38
Acute myocardial infarction	Other forms of chronic ischemic heart disease	2.47	Disorders relating to short gestation and unspecified low birthweight	Respiratory distress syndrome	2.51	Fracture of base of skull	Fall on or from stairs or steps	3.37

### Pairwise result

From the same MIMIC dataset, the pairwise method identified 10,109 associations (edges) among 589 diagnosis categories (nodes), and the edge list is presented in Supplementary material 5. It was challenging to interpret such a huge number of associations by the pairwise method. The most positive edge was between “effects of reduced temperature” and “excessive cold,” with a logOR of 10.71. At the same time, the most negative edge was between “disorders of lipoid metabolism” and “single liveborn,” with a logOR of −7.80. These associations appeared less insightful in the critical care setting.

### Simulation

Table 5 presents the simulation result. When sparsity was low (0.05), the pairwise OR method and graphic model methods performed similarly; the graphic model was a little better in Euclidean distance and Netsmile, while pairwise OR was better in spectral distance. However, when sparsity became high (0.1, 0.2), the pairwise method degenerated rapidly. The distance between the estimated graph and the original graph by the pairwise OR method became extremely high to hundreds or thousands, while the distance by graphic models remained low. For example, at a sparse rate of 0.1, a sample of 10k, pairwise OR was 292.47 in Euclidean distance, 2,638.95 in spectral distance, and 28.58 in Netsmile, while the best graphic model was 14.35 in Euclidean distance, 17.75 in spectral distance, and 9.41 in Netsmile. At a sparse rate of 0.2, a sample of 10k, pairwise OR was 968.46 in Euclidean distance, 9,296.11 in spectral distance, 31.12 in Netsmile, while the best graphic model was 50.96 in Euclidean distance, 62.45 in spectral distance, and 18.30 in Netsmile. The performances of graphic models using different percentages of the Lasso penalty were overall similar. In more sparse cases, a higher percentage of Lasso performed a little better, while in less sparse cases, a lower percentage of Lasso performed a little better, but they were overall similar. As a result, 90% Lasso elastic net was used as our graphical modeling method penalty. Our graphical modeling method also demonstrated superior performance on small networks when compared with pairwise OR and mutual information networks, see Supplementary material 6.

**Table 5:** Comparison of different methods in simulation studies.

**Sparsity**	**Sample size**	**Method**	**Euclidean distance**	**Spectral distance**	**Netsmile**
0.05	5k	Pairwise OR	11.52	**7.10**	14.29
		Elastic 100% LASSO	**11.00**	16.45	12.16
		Elastic 90% LASSO	11.12	16.77	**12.05**
		Elastic 70% LASSO	11.42	17.57	11.86
	10k	Pairwise OR	9.99	**5.48**	14.63
		Elastic 100% LASSO	**8.28**	12.14	10.12
		Elastic 90% LASSO	8.34	12.37	10.08
		Elastic 70% LASSO	8.55	12.95	**9.84**
0.1	5k	Pairwise OR	292.79	2,640.70	28.38
		Elastic 100% LASSO	17.38	**22.24**	**10.28**
		Elastic 90% LASSO	**17.33**	22.27	10.85
		Elastic 70% LASSO	17.76	23.44	13.33
	10k	Pairwise OR	292.47	2,638.95	28.58
		Elastic 100% LASSO	**14.35**	**17.75**	**9.41**
		Elastic 90% LASSO	14.39	18.18	10.59
		Elastic 70% LASSO	14.87	19.50	13.18
0.2	5k	Pairwise OR	964.53	9,270.28	30.92
		Elastic 100% LASSO	55.31	92.53	21.47
		Elastic 90% LASSO	52.47	**61.20**	**19.99**
		Elastic 70% LASSO	**51.79**	62.02	20.87
	10k	Pairwise OR	968.64	9,296.11	31.12
		Elastic 100% LASSO	54.42	85.22	20.70
		Elastic 90% LASSO	51.58	**62.45**	**18.30**
		Elastic 70% LASSO	**50.96**	65.34	19.31

Best performance metrics were highlighted in bold for each loss and each simulation scenario.

## Discussion

Our graphical modeling method was successfully applied in a CCU EHR to identify disease associations, demonstrating that our method is valid, useful, and widely applicable in clinical settings. Compared with the pairwise method, the graphical modeling method identified much fewer edges in the MIMIC dataset (10,109 vs. 2,806). This indicated that graphical modeling method ruled out many superficial/indirect associations that the pairwise method would have kept. Although some authors (Brunson et al., 2020) suggested pairwise methods were generally robust enough, our multivariable method outperformed pairwise methods in the simulation study, especially when the sparse rate was high.

The graphical modeling method was widely used in psychological studies (Dalege et al., 2017; Epskamp et al., 2018), and it used the LASSO penalty as a regularization method. In this study, we improved this method with the elastic net penalty and applied it to EHR. In EHR settings, usually, multiple covariates are strongly correlated both with each other and the outcome. In this scenario, LASSO would choose only one covariate indifferently, while elastic net (Zou and Hastie, 2005) would keep all the correlated covariates, generating a more realistic result. Because of this, elastic net improved the process of constructing a disease network in EHR compared with LASSO. Our method was also shown to perform better than the traditional method in a simulation study, especially when the sparsity rate was high. Ninety percent LASSO in the elastic net penalty was chosen as a convenient model, yet further studies were needed to fine-tune the LASSO percentage for a better fit of the model. In addition, patients' demographics were not included in the model to construct the comorbidity network. Future studies can improve the current model by adjusting patients' demographics when inferring the network.

Few studies have analyzed CNA in CCUs, where patients often have critical concomitant medical problems in multiple organ systems that may impact survival (Simpson et al., 2021). CNA could benefit CCU settings when a patient's status is often critical, and doctors need to diagnose and treat the patient immediately. Within this setting, having a valid comorbidity network could ensure that all likely comorbidities of a patient are identified and addressed, which could ultimately improve morbidity and mortality.

By examining the diagnosis categories with the highest positive and lowest negative edges, we demonstrated that our method successfully identified associated comorbidities that make sense in the clinical setting of the CCU. For example, several of our top positive edges involved poisonings with suicide and self-harm; clinically, exposure to any poison is associated with attempted suicide in between 29.7 and 50.5% of cases (Gummin et al., 2019); thus, it makes sense that these were highly correlated in our dataset. Similarly, multifetal gestations, including twins and higher-order multiples, commonly result in preterm delivery (ACOG Practice Bulletins, 2021), findings that were also commonly correlated in our analysis. The diagnosis categories with the lowest negative edges make sense clinically—you cannot have both a single liveborn and a twin or other multi-gestational liveborn pregnancy.

In addition to identifying diagnosis categories commonly associated across all disease sites, our method was also able to examine top edges within specific modularity classes. As previously, we also found that highly correlated diagnoses are clinically valid, such as the association between rheumatic heart disease with mitral and aortic value disease (Marijon et al., 2012) in the cardiac modularity. Because much of intensive care is now segmented into subspecialized settings such as cardiac critical care, surgical intensive care, and neonatal intensive care, it is important to have analytic methods adaptable to both general and specialized units. For example, using our CNA method within a specialized cardiac ICU could identify not only diagnoses correlated with cardiac diseases but also non-cardiac diagnoses that providers should be aware of. Knowing which diagnoses are correlated could avert missed diagnoses and potentially translate into decreased morbidity and mortality for ICU patients (Silfvast et al., 2003; Combes et al., 2004; Pastores et al., 2007).

Our analysis of the top nodes in this dataset provides interesting and useful information. While several top nodes and modularity classes we identified are commonly found in ICU settings, such as electrolyte/fluid imbalance (Lee, 2010), anemia (Napolitano, 2004), and poisoning (Rasimas and Sinclair, 2017; Mégarbane et al., 2020), it is helpful from an institutional and health system perspective to identify the most common diseases to plan resource acquisition and management. For example, knowing that anemia is common might prompt an ICU or healthcare administrator to ensure that there is a functioning and well-stocked blood bank in their hospital, or seeing a rise in cardiac cases may mean that a hospital needs to hire more cardiologists. In addition, being aware of the most common or most commonly associated diseases could prompt more accurate coding and ensure correct medical billing within a hospital system by suggesting missed common diagnoses to providers. Avoiding missed diagnoses to increase billing in ICU settings has the potential to substantially increase hospital income (Hendershot et al., 2009).

Beyond making sense clinically, our analysis provides potentially novel diagnosis associations that could be used as hypothesis-generating ideas or prompt further investigations. For example, our result found that upper limb contusion and “agents primarily acting on the smooth and skeletal muscles and respiratory system causing adverse effects in therapeutic use” were highly correlated within the injuries modularity; investigators could examine which agents are associated with contusions, how they are causing contusions, and whether this could be prevented. Our study examined an EHR specifically within one CCU setting. Nevertheless, it could be equally applied to other EHRs or across EHRs, providing a larger dataset to look for novel diagnostic associations.

In addition to the importance of CNA, our research had several limitations. The first limitation was phenotyping. Phenotyping arises because the presence of a code does not mean the presence of a disease. For example, sometimes differential diagnosis tests require using a disease code for billing purposes, and there might be errors or typos when inputting the code. To address this limitation, further studies could validate the diagnosis information with support information, such as more than one code, in different types of encounters/claims separated by a certain number of days and having relevant procedure codes/treatment codes. Second, the association identified by CNAs is not causal. For example, the current method did not indicate if “diseases of mitral and aortic valves” cause “rheumatic heart disease” or vice versa. Clinical knowledge is needed to infer and validate the causalities; for example, clinical knowledge is needed to understand the association between “rheumatic heart disease” and “diseases of mitral and aortic valves.”

## Conclusion

Comorbidity networks are important in clinical practice. This study introduced and improved a graphical modeling method to infer comorbidity networks under the EHR settings. This method outperformed the traditional pairwise method in simulation. When applying this method in CCU settings, a reasonable comorbidity network was constructed, and its associations were consistent with current medical understanding. The comorbidity network method has the potential to help CCU doctors to diagnose patients faster and minimize missed diagnoses. Future studies can be done to fine-tune the LASSO percentage to better fit the model or include patients' demographics in the model to construct an adjusted network.

## Data availability statement

Publicly available datasets were analyzed in this study. This data can be found at: https://physionet.org.

## Author contributions

XL, BZ, SR, and KF designed the study. BZ and GZ performed the data analysis and the simulations. SH and BZ reviewed the study results from a clinical perspective and provided clinical insights. All authors discussed the results and contributed to the final manuscript.

## Supplementary material

The R codes for running and comparing different methods are available at: https://github.com/rluo/ehr_net.

Supplementary material 1 Prevalence of diagnosis categories: 654 diagnosis categories.

Supplementary material 2 List of associations between diagnosis categories: Our method identified 2,806 associations among 510 diagnosis categories.

Supplementary material 3 Histogram of the degrees of all diagnosis categories from our comorbidity network identified by our graphic modeling method.

Supplementary material 4 Histogram of the distances between all diagnosis categories from our comorbidity network identified by our graphic modeling method.

Supplementary material 5 List of associations between diagnosis categories by the pairwise method: it identified 10,109 associations among 589 diagnosis categories.

Supplementary material 6 Additional comparisons of different methods using simulation studies of a small network.
