# Supplementary material for: Comorbidity network analysis using graphical models for electronic health records
Source: Front Big Data. 2023 Aug 17;6:846202. doi: 10.3389/fdata.2023.846202 (PMC10470017; doi:10.3389/fdata.2023.846202)
Supplement: Supplementary material 1 — Prevalence of diagnosis categories: 654 diagnosis categories. [file Data_Sheet_1.zip › Supplementary file 6.docx]

| Sparsity | Sample size | Method | Euclidean Distance | Spectral distance | Netsmile |
| --- | --- | --- | --- | --- | --- |
| 0.05 | 5k | Pairwise OR | 1.02 | 1.14 | 17.89 |
|  |  | Pairwise MI | 4.63 | 6.92 | 22.85 |
|  |  | Elastic 100% Lasso | **0.68** | **0.94** | 14.49 |
|  |  | Elastic 90% Lasso | 0.70 | 0.98 | 14.49 |
|  |  | Elastic 70% Lasso | 0.75 | 1.06 | **14.41** |
| 0.1 | 5k | Pairwise OR | 2.06 | 2.43 | 16.14 |
|  |  | Pairwise MI | 6.77 | 10.61 | 20.57 |
|  |  | Elastic 100% Lasso | **1.03** | **1.43** | **10.41** |
|  |  | Elastic 90% Lasso | 1.07 | 1.49 | 10.42 |
|  |  | Elastic 70% Lasso | 1.14 | 1.62 | 10.42 |
| 0.2 | 5k | Pairwise OR | 4.11 | 5.17 | 14.53 |
|  |  | Pairwise MI | 9.84 | 17.14 | 17.30 |
|  |  | Elastic 100% Lasso | **2.10** | **2.95** | 9.17 |
|  |  | Elastic 90% Lasso | 2.13 | 3.03 | **9.08** |
|  |  | Elastic 70% Lasso | 2.23 | 3.24 | 9.29 |

Supplementary material 6. Comparison of different methods using simulation studies of a small network. Best performance metrics were highlighted in bold for each loss and each simulation scenario. The simulation settings were almost the same as Table 5 of the main text. The node size here was reduced to 20 in order to compute the pair-wise mutual information (Pairwise MI) method due to its computational cost.
