# Supplementary figures and images for: Comorbidity network analysis using graphical models for electronic health records
Source: Front Big Data. 2023 Aug 17;6:846202. doi: 10.3389/fdata.2023.846202 (PMC10470017; doi:10.3389/fdata.2023.846202)

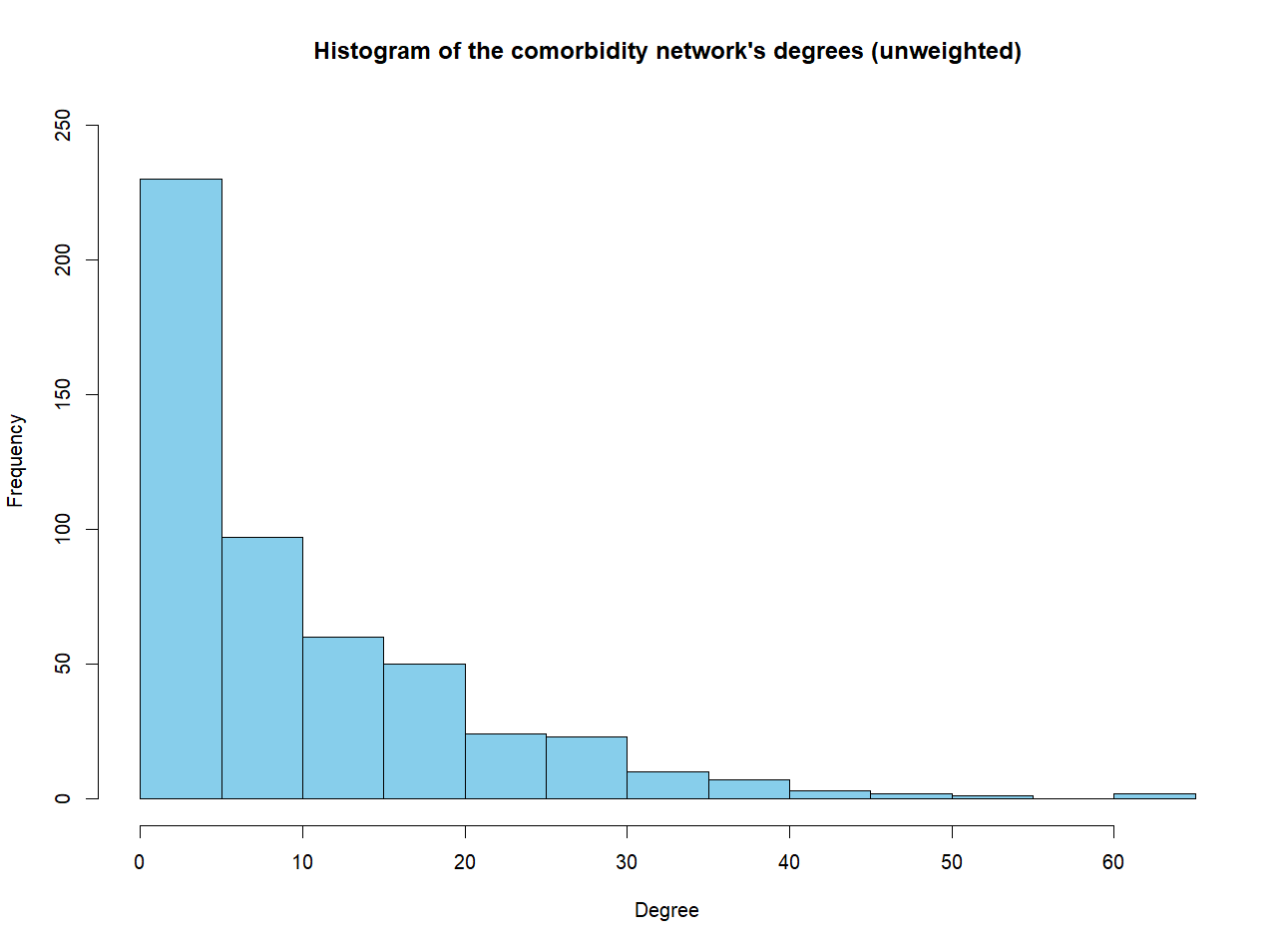

Supplement: Supplementary material 1 — Prevalence of diagnosis categories: 654 diagnosis categories. [file Data_Sheet_1.zip › Supplementary file 3.jpeg]

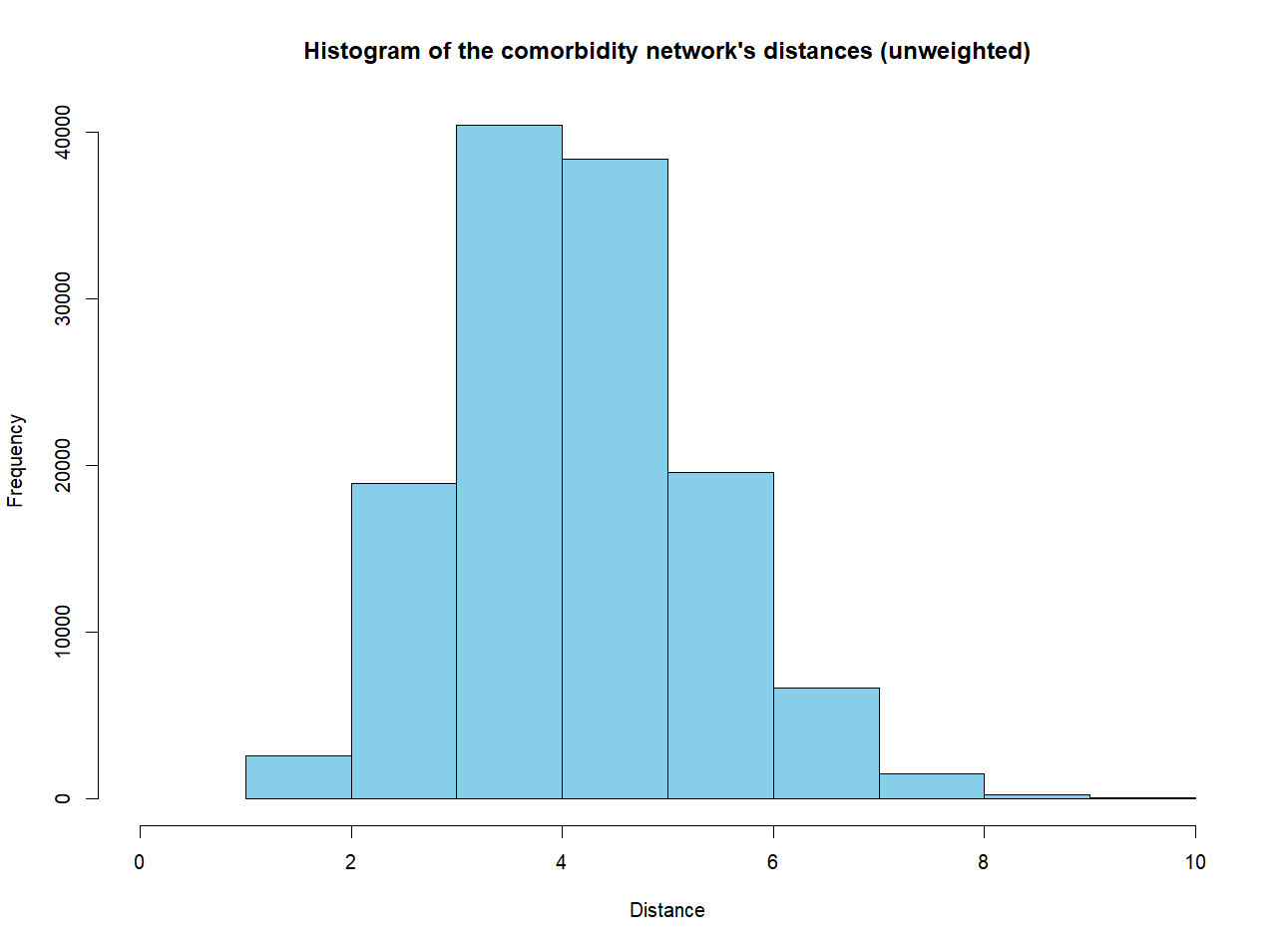

Supplement: Supplementary material 1 — Prevalence of diagnosis categories: 654 diagnosis categories. [file Data_Sheet_1.zip › Supplementary file 4.jpeg]
